# Adaptable and Robust EEG Bad Channel Detection Using Local Outlier Factor (LOF)

**DOI:** 10.3390/s22197314

**Published:** 2022-09-27

**Authors:** Velu Prabhakar Kumaravel, Marco Buiatti, Eugenio Parise, Elisabetta Farella

**Affiliations:** 1 Digital Society Center, Fondazione Bruno Kessler, 38123 Trento, Italy; 2 Center for Mind/Brain Sciences, University of Trento, 38068 Rovereto, Italy

**Keywords:** EEG, artifacts, local outlier factor, bad channels

## Abstract

Electroencephalogram (EEG) data are typically affected by artifacts. The detection and removal of bad channels (i.e., with poor signal-to-noise ratio) is a crucial initial step. EEG data acquired from different populations require different cleaning strategies due to the inherent differences in the data quality, the artifacts’ nature, and the employed experimental paradigm. To deal with such differences, we propose a robust EEG bad channel detection method based on the Local Outlier Factor (LOF) algorithm. Unlike most existing bad channel detection algorithms that look for the global distribution of channels, LOF identifies bad channels relative to the local cluster of channels, which makes it adaptable to any kind of EEG. To test the performance and versatility of the proposed algorithm, we validated it on EEG acquired from three populations (newborns, infants, and adults) and using two experimental paradigms (event-related and frequency-tagging). We found that LOF can be applied to all kinds of EEG data after calibrating its main hyperparameter: the LOF threshold. We benchmarked the performance of our approach with the existing state-of-the-art (SoA) bad channel detection methods. We found that LOF outperforms all of them by improving the F1 Score, our chosen performance metric, by about 40% for newborns and infants and 87.5% for adults.

## 1. Introduction

EEG is a widely used, non-invasive neuroimaging technique for recording the brain’s electrical activity for clinical monitoring, neuroscience research, and Brain–Computer Interface (BCI) applications [1,2]. However, the primary drawback of using EEG is its high susceptibility to biological and technical artifacts, i.e., signals that do not originate from the brain [3]. Common artifact sources include the electrical activity of the eyes, heart, and muscles, electrical artifacts due to cable movements, and electromagnetic interference from the surroundings [4].

To recover the neural information, such artifacts should be identified and removed from the acquired data. Several artifact removal methods based on the widely used Independent Component Analysis (ICA) have been proposed [5,6,7,8,9] that work best for stereotypical artifacts such as eye blinks. To deal with non-stereotypical artifacts, such as motion artifacts, the Artifacts Subspace Reconstruction (ASR) algorithm is increasingly becoming popular [10,11,12]. However, all these methods require a crucial, preliminary step: detecting and removing noisy sensors/channels. This work focuses on this important preprocessing step. EEG channels/sensors that have a poor signal-to-noise ratio (SNR) due to biological or technical artifacts contaminating a larger portion of the recording are commonly termed as “bad channels”. Bad channel detection is crucial in removing artifacts for the following reasons:

(i) Removing noisy segments of EEG in the presence of bad channels can lead to severe data loss due to a misleading overall rejection threshold.
(ii) The presence of bad channels can produce a strong bias on the overall statistics of the extracted neural features leading to the wrong interpretation of the experiments.
(iii) Further, bad channels can also bias the source level analysis as they often suppress the information from the adjacent good channels, resulting in a wrong source reconstruction.

The artifact preprocessing strategy varies depending on the population from which the EEG was acquired and the employed experimental paradigm. For example, in adult EEG, the artifacts have well-defined temporal and spatial features such as eye blinks (here, ICA is a good solution). Instead, developmental EEG collected from newborns, infants, or young children present more challenges in cleaning as the artifacts are primarily due to uncontrolled motion (here, ASR processing before ICA is recommended [13]). As such, artifact removal tools developed for adult EEG might not be optimal for newborn EEG. Secondly, EEG artifact preprocessing also depends on the experimental paradigm. For example, EEG offline preprocessing for computing Event-Related Potentials (ERPs) requires a different cleaning strategy compared to EEG processing for Frequency-Tagging methodology. This is because the neural response of the latter, being associated with a specific frequency instead of a broad frequency range, is less affected by artifacts than ERP responses [14]. In sum, the experts’ annotations of bad channels usually vary according to the population and the experimental design.

In a broad sense, bad channel detection is an anomaly detection problem. It is the process of finding records that significantly deviate from the regular data. Usually, the total number of anomalies is lower than the regular ones in a given dataset. Depending on the availability of labels for regular and anomaly data points, supervised (which requires labels for both classes), semi-supervised (which requires labels only for regular data points), and unsupervised (which requires no labels) methods can be employed. Here, we briefly discuss the state-of-the-art anomaly detection methods using unsupervised learning approaches.

Ramaswamy et al. proposed a *k*-nearest neighbors global anomaly detection method [15]. First, the number of *k* neighbors is assigned for the given data. Then, the distance to the *k*-th nearest neighbor is used to rank the outliers. The drawback of this approach is that the outliers close to the clusters are often undetected (as this technique is “global” rather than “local”) [16]. To overcome such a limitation of distance-based outliers detection, Breunig et al. were the first to propose the idea of a local anomaly detection algorithm: the Local Outlier Factor (LOF) [17]. The LOF score is the ratio of the local density of a record to that of its *k*-nearest neighbors. An interesting property of LOF is that the average regular instances with similar densities to their neighbors will have a score of 1.0. In terms of interpretability, the LOF score is better than the arbitrary score we achieve using the *k*-nearest neighbors method. Yet, it is not straightforward to establish a threshold for the LOF score that separates outliers from normal points. Authors in [18] attempted to overcome this difficulty by replacing the conventional LOF scores with an anomaly probability called Local Outlier Probability (LoOP). The LoOP algorithm computes the standard deviation of distances to the nearest neighbors based on the assumption that distances follow a half-Gaussian distribution. The resultant probabilistic set distance is used to compute the local density score, to which a Gaussian error function is applied to derive a final probability measure. Despite an interpretable probabilistic measure for anomalies, the approach received critical thoughts [19]. Notably, the authors in [16] showed that LoOP probability scores are equivalent to the normalized LOF scores (i.e., in the range [0, 1]).

In recent years, the scientific community has removed threshold subjectivity to outlier detection by incorporating outlier probabilities for structural health monitoring applications [20,21]. These methods are validated on 1-D point vectors where each point was assigned an outlier probability. In the context of EEG, however, if the total number of outlier points is identified for each channel, then a threshold is still required to determine the final set of anomalous channels. Further, it is worth noting that analyzing individual sensors might not produce desirable results in a multi-channel EEG where the spatial correlation between channels is a vital property. It is, therefore, essential to find the hidden local properties in the data from multiple sensors. Hence, it is not clear whether the methods proposed in [20,21] are suitable for the EEG bad channel detection problem.

Within the EEG literature, the widely used bad channel detection methods employ measures such as Kurtosis [22], Pearson Correlation [23], Channel Variance, Hurst Exponent [24], and Normalized Power Amplitude [25]. While these methods have shown remarkable results in their respective studies, there are mainly three limitations: (1) Most of these methods assume a normal distribution for EEG data and obtain distribution-based statistical measures (e.g., Kurtosis, Variance, Standard Deviation) to detect bad channels. However, most real EEG data do not follow a normal distribution unless the data length is extremely short [26]. (2) Measures such as Channel Variance do not consider the intrinsic variability of the EEG signal across channels (for example, the variance of EEG amplitudes in frontal electrodes is usually higher than the one from central electrodes). (3) These methods were validated on only one kind of EEG (i.e., measures used in [23,24] for adult EEG; measure used in [25] for infants EEG). Our preliminary analysis suggested that they produce sub-optimal results when applied to other kinds of data than the ones that they are intended for. In addition to these traditional methods, there are a few deep neural network-based approaches to detect anomalous EEG channels [27,28,29]. For the compactness of this paper, they are not discussed in further detail as we focus only on traditional models.

Given the importance of identifying local patterns in EEG bad channel detection, in this work, we consider the Local Outlier Factor (LOF) [17,30] to automatically detect and remove bad channels. LOF is a “local” approach because it measures the degree of isolation of a given channel with respect to its “local” neighborhood (where the neighborhood is defined using the *k*-neighbors algorithm [31] computed from the activity vectors associated with each channel and not to be confused with the spatial distance between the electrodes). In other words, LOF assigns an outlier score for each channel by computing its local density, where locality is defined by the *k*-neighbors algorithm. Thanks to this property, LOF is a robust technique compared to traditional methods that employ global measures of uncertainty and, therefore, is adaptable to the differences in the EEG mentioned above.

As we mentioned earlier, it is not straightforward to find a decision threshold that separates outliers using LOF scores. This decision boundary depends on the nature of the data and the definition of outliers. In this work, we propose an automated calibration approach to identify the optimal threshold using a pre-labeled dataset collected from the same laboratory and under a similar experimental setup. The proposed approach is based on our observation that the optimal LOF threshold does not change from one dataset to another given similar EEG acquisition settings (i.e., same EEG system, similar population, and same experimental design).

In previous work, we introduced LOF for the first time on EEG data as the first step of a pipeline for artifact removal in developmental studies [13]. Here, we provide a complete characterization of LOF, presenting further development and validation of the method in the following three directions: (1) We present a novel, robust, and fully automatic method for computing LOF key parameters from a single dataset with annotated bad channels; (2) To test LOF adaptability to any kind of data, we validate LOF on newborn, infant, and adult datasets. We highlight that each of these datasets was acquired using different EEG paradigms; (3) To prove the robustness of the proposed approach, we systematically compare LOF performance with other EEG bad channel detection measures.

As a proof-of-concept, we first validated LOF on simulated EEG generated with the SEREEGA toolbox [32] and contaminated five randomly chosen channels with different kinds of artifacts. With the known ground truth, we validated the performance of LOF. Then, we validated LOF on real EEG datasets acquired from three different populations: newborns, infants, and adults. The newborn datasets (*n* = 21) with annotated bad channels were acquired in the study [14]. For infants, we used EEG (*n* = 28) acquired in another study [33] with annotated bad channels via visual inspection by the respective authors. We used the open-source adult datasets (*n* = 14; multiple sessions for each participant leading to an overall 113 files) with annotated bad channels from OpenNeuro [34].

For comparative evaluation, we chose state-of-the-art (SoA) methods such as Kurtosis and bad channel detection techniques in widely used EEG pipelines, namely, FASTER [24,35], CRD [23,36], and HAPPE [25]. Considering the imbalanced proportion of good and bad channels (94% vs. 6%), we validated all methods using a robust metric: the F1 Score [37]. This is the first study to evaluate and compare bad channel detection methods on EEG acquired from different populations. The source code compatible with EEGLAB [22] is made freely available (https://github.com/vpKumaravel/NEAR/tree/main/NEAR_ChannelRejection-master (accessed on 11 August 2022)) [38].

## 2. Materials and Methods

### 2.1. LOF Algorithm

**Figure 1:**
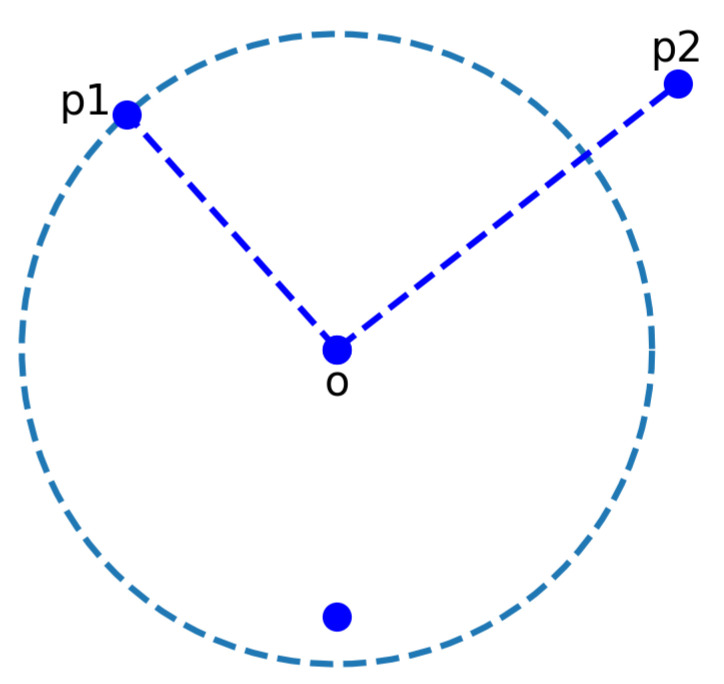
An example scenario for the computation of reachability distance using *k* = 3. The dotted circle represents the *k* neighborhood of point *o*. All blue points represent the data samples. For the demonstration, let us consider only two points, *p1* (lies within the *k* neighborhood) and *p2* (lies outside the *k* neighborhood). The reachability distance between point *p1* and *o* will be the *k*-distance (*knnsearch*, MATLAB [40])) whereas the reachability distance between point *p2* and *o* will be the Euclidean distance between them.

The LOF algorithm quantifies the outlierness of each electrode in the multidimensional activity space where each electrode is associated with a vector representing its EEG activity (not to be confounded with its physical location on the scalp). The algorithm is described as follows:

1. The optimal *k* value (i.e., the number of nearest neighbors) is first computed using the Natural Neighbors algorithm (NaN [39]), a data-centric non-parametric approach.
2. For a given channel *p*, the LOF algorithm identifies *k* neighbor channels based on the predefined distance metric (e.g., Euclidean) using the *k*-nearest neighbors algorithm [31].
3. Then, a reachability distance is computed between channels. For example, let us consider two channels, namely *p* and *o*. The reachability distance between *p* and *o* is computed as follows:
  (1) $$\mathrm{reach-dist}{\;}_{k}\left(p,o\right)=\max\left\{k\mathrm{-distance}\left(o\right),d\left(p,o\right)\right\}$$
  where *k*-distance (*o*) is computed using the *knnsearch* function (MATLAB [40]) and d(p,o) is the Euclidean distance between two channel vectors. Intuitively, if channel *p* is far from *o*, the reachability distance is their actual Euclidean distance. Instead, if they are sufficiently close, the Euclidean distance is replaced by the *k*-distance of channel *o* (See Figure 1). Considering the *k*-distance rather than the actual distance reduces the statistical fluctuations for the points existing within the *k* neighborhood.
4. Once the reachability distance of each channel with respect to its neighbors is computed, then the local reachability density (LRD) is determined as follows:
  (2) $${\mathrm{LRD}}_{k}\left(p\right)=1/\left(\frac{\sum_{o\in N_{k}\left(p\right)}\;\mathrm{reach-dist}{\;}_{k}\left(p,o\right)}{\left|N_{k}\left(p\right)\right|}\right)$$
  where $N_{k}\left(p\right)$ refers to the total number of *k* neighbors of *p*.
  To put it in words, the LRD of the channel *p* is the inverse of the average reachability distance based on the k-nearest neighbors of *p*. Intuitively, channel *p* will have a lower LRD if it were an outlier (i.e., bad) channel because it is not easily "reachable" by most of its neighbors.
5. As a final step, the local outlier factor (LOF) is computed as follows:
  (3) $${\mathrm{LOF}}_{k\;}\left(p\right)=\frac{\sum_{o\in N_{k\;}\left(p\right)}\frac{{\mathrm{LRD}}_{k\;}\left(o\right)}{{\mathrm{LRD}}_{k\;}\left(p\right)}}{\left|N_{k\;}\left(p\right)\right|}$$
  The LOF of channel *p* is the ratio of the average LRD of *k* neighbors of *p* to the LRD of *p*. The lower *p*’s LRD is, and the higher the LRD of *p*’s k-nearest neighbors are, the higher the LOF value of *p* is (and, therefore, possibly an outlier). In other words, an outlier channel would display a lower LRD (therefore, larger in distance) compared to its neighbors (on average). Note that if channel *p* has a similar LRD value compared to its *k* neighbors, the LOF score would be approximately 1.

### 2.2. LOF Threshold Computation

In an ideal scenario where the objects (or samples) form a uniform or a Gaussian cluster, inliers would yield LOFs approximately equal to 1, as can be inferred from Equation (3). As such, any object (or sample) that exceeds a LOF score of 1 can be considered an outlier. However, this criterion might vary in real-world data, where the distribution of objects is unknown and less likely to be uniform or Gaussian. A thorough investigation of the decision boundary is required as there are different EEG settings (populations, experimental paradigms, and so on) and the definition of outliers varies according to the settings. Therefore, in this work, we consider the LOF_thr_ as a hyperparameter to be optimized using the supervised approach (i.e., with annotated bad channels as the true labels). Precisely, we used the k-fold cross-validation technique [41] to systematically identify the optimal LOF_thr_ (exhaustive search in the range between 1 and 5, in steps of 0.1) at which the F1 Score is maximized.

(4) $$\;F1\;\mathrm{score}\;=\frac{2\times \mathrm{TP}}{2\times \mathrm{TP}\;+\;\mathrm{FP}\;+\;\mathrm{FN}}$$

where TP, FP, and FN indicate the number of true positives, false positives, and false negatives respectively.

**Figure 2:**
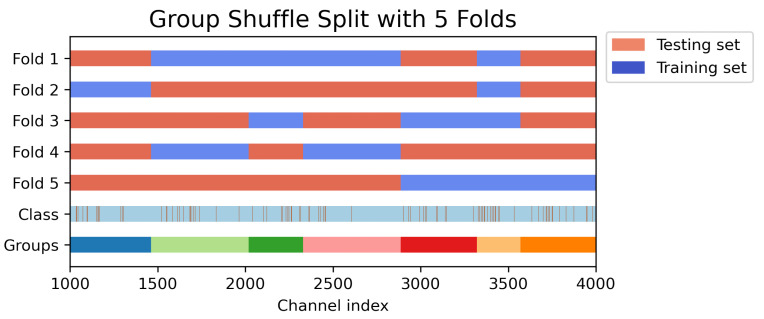
A working example of the group shuffle split cross-validation technique with test size = 50%. The *x*-axis represents the channel indices. The brown vertical lines in the ‘Class’ row indicate bad channels while the blue background represents good channels. ‘Groups’ indicate the subjects, each containing a different number of sessions. For illustration purposes, we restricted the number of folds to five and the number of subjects (‘Groups’) to seven. Note that in any given fold, no group simultaneously takes part in both training and test sets, thereby avoiding subject-specific data leakage.

We used the number of folds k = 10, a common choice in machine learning [42,43], and for each fold, 50% of the data are used for testing on both newborns and infants datasets. As our adult dataset contains multiple sessions from the same subject, and in order to avoid subject-specific leakage in the training set, we used the group shuffling procedure (using the GroupShuffleSplit method from SciKit [41]) rather than using the default random shuffling in each fold. An example is shown in Figure 2. For visualization purposes, we show only five folds and seven groups (i.e., seven subjects with a diverse number of sessions each). The ‘class’ label indicates two classes: good and bad channels (indicated as vertical lines in orange). The ‘Groups’ label shows different colors for each subject, and the number of channels in each group varies (depending on the number of EEG recording sessions for each subject). It can be seen that the groups used as the training set for a particular fold are not used as the testing set (thereby avoiding data leakage), and in each fold, different combinations of groups are used for training to effectively validate LOF on the limited EEG samples (113 files with 62 channels each leading to a total of 7006 EEG channels).

In addition, since LOF scores can be different depending on the employed distance metric, we compared the classification performance of LOF using each of these two metrics: Euclidean (*euc*; ‘euclidean’ in MATLAB [40]) and Standardized Euclidean (*seuc*; ‘seuclidean’ in MATLAB [40]). As the other possible distance metrics, such as ‘correlation’ and ’spearman’, performed worse in our empirical analysis, we discarded them for further analysis. To provide a quick review for the readers, here we define Euclidean (*euc*) and Standardized Euclidean (*seuc*) of two point vectors *p* and *q* with cardinality *n*. The Euclidean distance is the length of a line segment between two points in Euclidean space and is defined as

(5) $$euc\left(p,q\right)=\sqrt{\sum_{i=1}^{n}{\left(q_{i}-p_{i}\right)}^{2}}$$

The Standardized Euclidean distance (*seuc*) is, in essence, the Euclidean distance computed using standardized data (i.e., each coordinate difference is scaled by the corresponding standard deviation) defined as

(6) $$seuc\left(p,q\right)=\sqrt{\sum_{i=1}^{n}{\left(\left(q_{i}-p_{i}\right)/std\left(q_{i},p_{i}\right)\right)}^{2}}$$

### 2.3. Bad Channel Detection based on Statistical Measures

The simplest features to detect bad channels are channel-wise mean amplitude (Mean), Inter-Quartile Range (IQR), or Median Amplitude Deviation (MAD). The outliers in the data influence the Mean, IQR, and MAD values (even if IQR and MAD are more robust measures than the Mean). As such, the thresholds that work well for noisier data cannot be optimal for relatively cleaner data and vice versa.

As LOF measures the degree of outlierness by considering only the cluster of neighboring channels and not the whole distribution of the data, the obtained LOF scores are relatively insensitive to outliers present in the data compared to the aforementioned features. To numerically validate this statement, we computed the Mean, IQR, and MAD for each channel of all EEG files. Further, we computed LOF scores using both Euclidean (*euc*) and Standardized Euclidean (*seuc*) as distance metrics for comparison. Each measure (e.g., Mean) from each EEG file is normalized to keep the [0, 1] range. Then, we changed the decision threshold from 0 to 1 in steps of 0.005 and computed the False Positive Rate (FPR, i.e., the probability of inaccurately predicting the “good” channel as “bad”) and True Positive Rate (TPR, i.e., the probability of accurately predicting “bad” channel as “bad”) for each threshold. An aggregate measure AUC (Area Under the Curve), which uses both FPR and TPR is used as the validation metric. The feature with the highest AUC value can be considered optimal for bad channel detection.

### 2.4. State-of-the-Art Bad Channel Detection Methods

In this section, we introduce the state-of-the-art methods for detecting bad channels in EEG that we will compare with LOF.

1. Kurtosis
  Kurtosis is a higher-order statistical measure that reflects the Gaussianity of a distribution. Positive kurtosis indicates a super-Gaussian distribution, while negative kurtosis denotes a sub-Gaussian distribution. Despite being a simple measure, it has been widely used as a reliable feature for several artifact removal methods in EEG [44,45,46]. We used the EEGLAB function *pop_rejspec* to detect bad channels with default parameter settings. In particular, the kurtosis values computed for each channel were normalized to have zero mean and unit standard deviation (using z-score). Channels with a z-score of more than five were identified as bad channels.
2. FASTER
  FASTER is an automatic EEG artifact rejection method based on statistical thresholding [24]. FASTER detects bad channels using the following features: (i) Inter-channel Correlation Coefficient, (ii) Channel Variance, and (iii) Hurst Exponent [47,48].
3. Clean Raw Data (CRD)
  EEGLAB offers an automated approach to clean continuous raw EEG data using the Clean Raw Data (CRD) plugin [36]. CRD first looks for “Flat-Line” channels (i.e., channels that recorded constant values for at least 5 seconds). Then, it looks for bad channels that had predominantly recorded power-line interference noise, and finally, it looks for spatially uncorrelated channels.
4. HAPPE
  While all the above-mentioned techniques were developed for adult EEG, the HAPPE pipeline is one of the first preprocessing pipelines for removing artifacts from pediatric EEG [25]. In such data, the level of noisiness is comparatively higher and difficult to process. To detect bad channels, HAPPE uses the joint probability measure of the average log power computed between 1 and 125 Hz across all channels. Precisely, channels are predicted as bad if the computed probability falls more than three standard deviations from the mean. Since developmental EEG presents severe contamination of artifacts compared to adult EEG, the authors performed the computations twice for each file.

## 3. Description of EEG Datasets

### 3.1. Simulated EEG

As a proof-of-concept, we first validated LOF on simulated neurophysiologically plausible EEG data with known ground truth for bad channels by using the toolbox SEREEGA [32] along with our custom scripts to contaminate arbitrarily chosen channels. Precisely, we generated Steady-State Visually Evoked Potential (SSVEP) data with 64 channels using the following components (See Appendix A for more details):

Component 1: An SSVEP response with a stimulation frequency of 0.8 Hz was added in bilateral sources in the early visual cortex (MNI coordinates: [−8 −76 10] and [8 −76 10]).

Component 2: Event-unrelated ongoing EEG activity was generated in 62 randomly selected cortical sources, plus in the 2 sources of the first component located in the early visual cortex. Such activity is generated as Brown noise (power spectrum increasing as $1/f^{2}$ for f→0), mimicking the one observed in newborns [49]. Importantly, the signal-to-noise ratio between component 1 and component 2 was of the same order of magnitude as the one measured on real, artifact-free EEG data.

**Figure 3:**
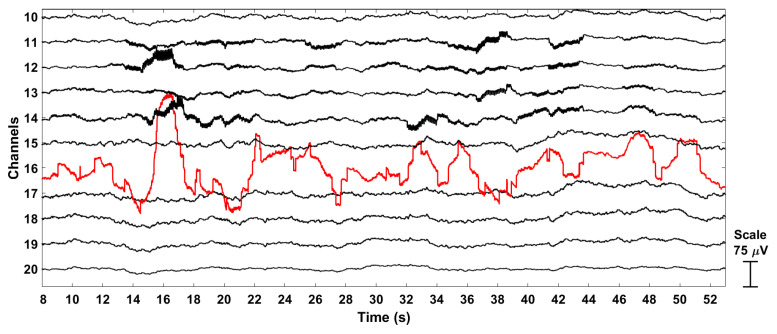
A sample portion of the simulated EEG. Bad channel 16 (in red) is contaminated with aperiodic, step-like artifacts. The channels in black are good channels.

Component 3: Once the neural signal was generated, artifacts in five randomly chosen channels were added, consisting of intermittent potential shifts and flat signals mimicking electrical discontinuities, and low-frequency fluctuations (0–10 Hz) mimicking local bad contacts and movement artifacts. Specifically, flat signals of constant amplitude were assigned to channels 1 and 49; channels 6 and 35 were contaminated with motion noise; and channel 16 was contaminated with aperiodic artifacts, representative of jump-like artifacts (see Figure 3).

### 3.2. Newborn EEG

We used the datasets collected from two different studies: (a) 10 healthy human newborns with a mean age of 60 ± 22 h for the study investigating face perception in newborns using the Frequency-Tagging paradigm [14], and (b) 11 healthy newborns with a mean age of 40 ± 16 h for another study investigating the neural basis of number perception in newborns (Buiatti et al., in preparation). Both datasets were acquired using an EGI amplifier (GES 400, Electrical Geodesic, Inc, Eugene, OR, USA) at a sampling rate of 250 Hz, referenced to the vertex. We applied a low-pass FIR filter with a cut-off frequency of 40 Hz to the raw data. Subsequently, we applied a non-causal high pass filter with [0.1 0.35] Hz as the transition band and a stop-band attenuation of 80 dB. Channels were marked as bad by the authors of the respective studies using a semi-automated approach (i.e., using the TrimOutlier toolbox [50] and visual inspection of time course and frequency distribution). The resultant bad channels are considered as ground-truth in this work. We highlight that the annotation of bad channels was carried out before the publication of the original study [14].

### 3.3. Infant EEG

We used the datasets from a study investigating semantic understanding of common nouns in preverbal 9-month-old infants [33] using the Event-Related Potentials (ERP) paradigm. All 28 infants were born full term (gestational age: 37 to 41 weeks) in the normal weight range (>2500 g). The datasets were acquired using an EGI amplifier (GES 300, Electrical Geodesic, Inc., Eugene, OR, USA) at a sampling rate of 500 Hz with a low-pass filter at 200 Hz. Continuous EEG was recorded by 125-channel Geodesic Sensor Nets referenced to the vertex. All EEG data were visually inspected for bad channels by the original study’s authors, which are considered the ground truth in this work. Again, the annotation of bad channels was carried out before the publication of the original study [33].

### 3.4. Adult EEG

We used the data from the study [51], validating alpha-power lateralization as feedback to enhance the visual covert attention task. A total of 14 subjects with a mean age of 23 years took part in the recordings on three different days, resulting in 130 EEG files (refer to [51] for more details related to the experimental setup). EEG was recorded with a 64-channel HIamp EEG system (g.tec, Austria) at a sampling rate of 512 Hz. The electrodes were positioned in the standard international 10-10 system. All datasets are available on the OpenNeuro platform [34]. Out of 130 files, only 113 were usable, and the others were found corrupted due to import issues. Before applying the LOF algorithm, we filtered the data at 40 Hz to remove the high-frequency noise components, and subsequently, a high-pass filter was applied to remove DC drifts. The ground truth bad channels are labeled by visual inspection by the original study’s authors and indicated as "bad" in the channel description for each EEG file on the OpenNeuro platform.

## 4. Results

### 4.1. LOF vs. Statistical Measures

**Figure 4:**
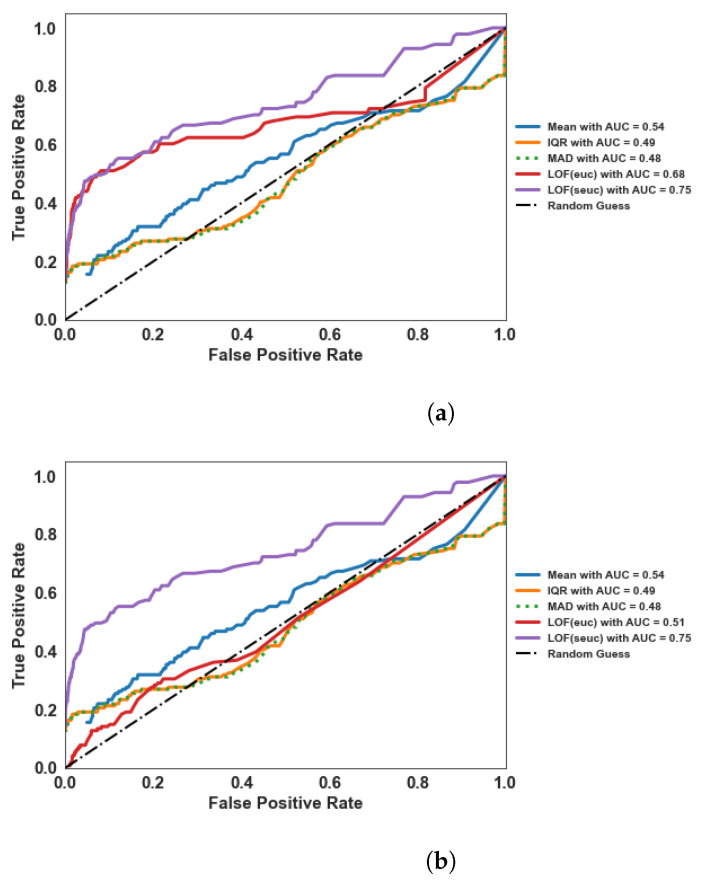

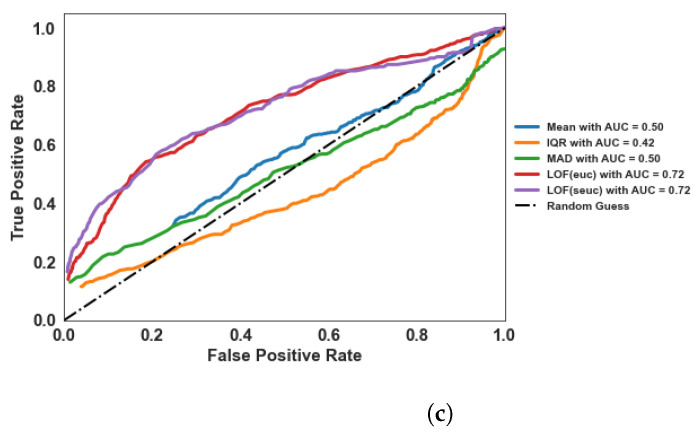
Robustness of LOF algorithm compared to the mean and the median-based (namely, Interquartile Range (IQR) and Median Absolute Deviation (MAD)) techniques in detecting bad channels in (**a**) newborn, (**b**) infant, and (**c**) adult data. For comparison within LOF, we used the default Euclidean distance metric (*euc*) and the Standardized Euclidean metric (*seuc*). LOF (*seuc*) performs better than LOF (*euc*) and the considered statistical measures.

We compared the classification performance of statistical measures such as Mean, IQR, and MAD as well as the LOF using Euclidean (*euc*) and Standardized Euclidean (*seuc*) distance metrics by performing the Area Under the Curve (AUC) analysis. The results are presented in Figure 4. For all three populations, we observed remarkable improvement in performance (the AUC curves are concave) for both variants of LOF compared to all other measures. This suggests that the LOF score is a robust measure against existing outliers in the data compared to Mean, IQR, and MAD. Further, we observed that LOF using the *seuc* metric outperformed the LOF using the *euc* metric for newborns and infants datasets, while for the adults dataset, both metrics achieved similar performance. For the analysis presented hereafter, we based LOF computation on the *seuc* metric.

### 4.2. Simulation EEG

We first validated LOF on a synthetic EEG dataset with known bad channels as a proof-of-concept. The results obtained from the simulated data are summarized in Table 1. While all methods detect the high-frequency muscle artifacts, only Kurtosis and LOF succeeded in catching the aperiodic, step-like artifact channel (ID: 16). It is worth highlighting that Kurtosis classified two good channels as bad (i.e., false positives) while LOF had no false positives. However, we observed that LOF does not detect the flat line channels. To deal with this, we integrated the flat line detector (used in the CRD toolbox [23]) with LOF in our tool [13], resulting in an F1 Score of 1. We, therefore, recommend applying a flat line detector prior to LOF to obtain the best results. To understand the influence of the number of channels in the EEG system, we simulated data with 32 and 16 electrodes using the same strategy. LOF produced similar results (i.e., F1 Score of 1) with 32-channel simulated EEG, and a slight performance degradation was observed (F1 Score of 0.89) with 16-channel EEG, still outperforming comparative methods.

### 4.3. Real EEG

**Figure 5:**
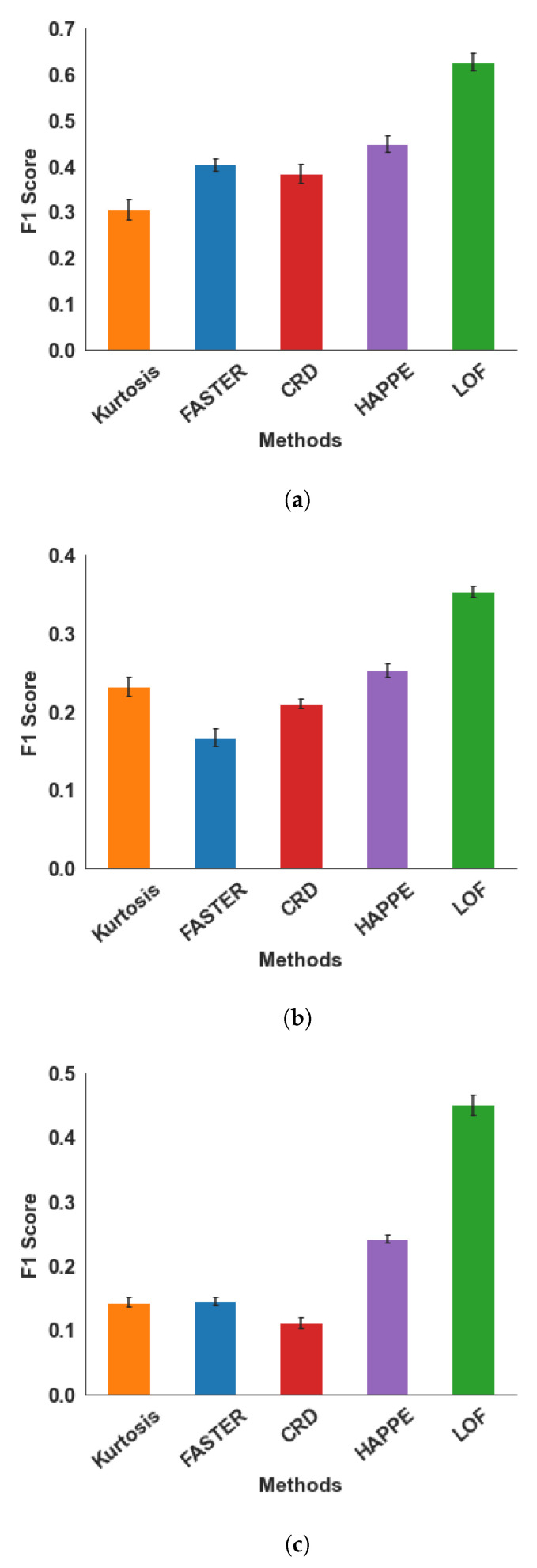
Performance of bad channel detection methods using the 10-fold cross-validation technique on (**a**) Newborn, (**b**) Infant, and (**c**) Adult EEG. The error bars represent the s.e.m. across validation folds.

We performed 10-fold cross-validation [42] for each population dataset (with group shuffling [41] for adult data and random shuffling for infants and newborns data), and the average F1 Score across all folds is summarized in Figure 5. The numerical values are also reported in Table 2.

LOF unequivocally outperformed the other methods in all kinds of data, proving its robustness to different SNR ranges of real data obtained using distinct experimental paradigms. For newborns (Figure 5a) and infants (Figure 5b), we observed improved performance of up to 40% compared to other SoA methods. For adults (Figure 5c), an improvement in performance up to 87.5% was observed.

### 4.4. LOF Optimal Threshold

**Figure 6:**
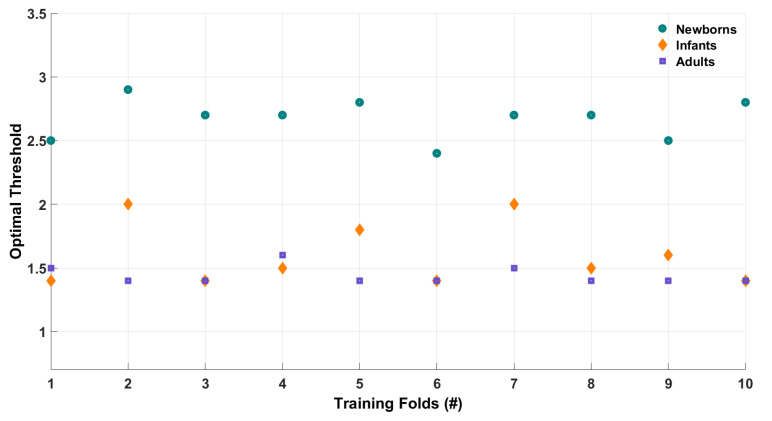
Summary of optimal range of LOF_thr_ for different populations. For newborns (low SNR data), a relaxed threshold of 2.6 is optimal, whereas, for infants (better SNR data), a value of 1.6 is found to be optimal. Finally, for adults (high SNR data), a conservative threshold of 1.4 is optimal.

We then investigated how the optimal LOF_thr_ varies within and across populations by using 10-fold cross-validation (see Figure 6). For newborns (noisy data, frequency-tagging paradigm), on average, the optimal threshold was identified as 2.6±0.16. For infants (mildly noisy data, ERP paradigm), it was 1.6±0.24. For adults (relatively clean data, event-related design with spectral power analysis), a further relaxed threshold of 1.4±0.07 was identified to be optimal.

## 5. Discussion

Most current bad channel measures rely on distribution-based statistics (Mean, Variance, Kurtosis). The primary drawback of such measures is that the underlying EEG data distribution is not purely Normal/Gaussian. Therefore, fitting the data into such standardized distributions might not produce satisfactory results. Further, these methods have been calibrated and validated on only one kind of EEG (i.e., either adult EEG or infant EEG). Given the differences in the EEG distribution according to the population and experimental design, these measures might not be reliable for other kinds of EEG than the ones they are intended for. This work introduced a unique, robust measure (Local Outlier Factor) for detecting bad channels adapted to EEG acquired in any setting.

To better understand why and under what conditions LOF works, we simulated realistic EEG with known bad channels. We showed that LOF efficiently captures the non-stereotypical motion artifacts differently from other methods while simultaneously keeping false positives to a minimum. The only limitation is that LOF fails to detect the flat-line channels. Therefore, we recommend that readers use a flat-line detector [36] before LOF analysis for better results.

LOF is an unsupervised outlier detection originally proposed for suspicious activity detection in Knowledge Discovery in Database (KDD) applications. However, there is no clear indication of which should be the decision threshold to detect outliers. In theory, a data object (in our case, an EEG channel) is an outlier if it has a LOF score of more than 1.0. Our preliminary analysis showed that this threshold is too strict (resulting in higher false alarms) for EEG data, which motivated the need to find the optimal LOF threshold. In this study, we showed how to find the optimal LOF threshold using a single dataset (employing a 10-fold cross-validation) to get the best results. Our analysis notably suggested that an optimal threshold for LOF lies around 2.5 for noisier data (newborns EEG) and approximately 1.5 for relatively cleaner data (infants and adults EEG). We strongly recommend the users follow a similar procedure to calibrate the LOF threshold for their own data. Precisely, we suggest the users take a portion of datasets to be analyzed (or previously collected datasets using similar EEG settings) and visually inspect for the bad channels to calibrate the LOF threshold. In cases where it is impossible (due to the unavailability of labeled data), we suggest an initial threshold of 1.5 for infant and adult EEG and 2.5 for newborn EEG based on our study results. In the future, it is desirable to have variants of LOF or other local outlier detection algorithms without the subjectivity of the decision threshold. As such, it is worth investigating the algorithms proposed in [20,21] for bad channel detection.

Another hyperparameter we considered is the distance function that LOF utilizes to compute the local density. We analyzed four possible metrics, namely: Euclidean, Standardized Euclidean, Pearson Correlation, and Spearman Correlation, and we found the latter two metrics yielded worse results and discarded them from further analysis. Among the Euclidean metrics, the Standardized Euclidean (*seuc*) performed better than the Euclidean distance (*euc*). This comparison suggests that for reliable bad channel detection using LOF, the direction of electrical activity plays a more critical role than the magnitude. It is a desirable property of LOF applied to EEG as the intrinsic amplitude fluctuations (due to sensor location and EEG oscillations [52]) do not impact the outlier detection.

Since LOF is a density-based approach, we also investigated the influence of the number of channels on the algorithm’s performance. Our empirical results in both simulated and real adult EEG data suggest that LOF is suitable for high-density EEG setups with at least 32 channels. Therefore, we recommend that users do not use the proposed approach on low-density EEG (i.e., less than 32 channels). Further developments in the future are required to make LOF suitable even for low-density EEG.

Another desirable property of LOF is that it does not assume any distribution for the raw EEG data. The LOF measure is loosely coupled to clustering algorithms (such as *k*-nearest neighbors algorithm [31]) and is computed using the relative density of the identified clusters [17]. Thanks to this, LOF is adaptable to EEG acquired in different settings. Further, the LOF score is comparatively robust to outliers in the data, as shown in Figure 4a. With optimal parameters, LOF succeeded in detecting the annotated bad channels compared to the traditional methods, such as Kurtosis, FASTER, and CRD, which assume a normal distribution for the EEG signal.

Remarkably, the second-best performing algorithm was HAPPE [25] with all datasets. This merit is likely because HAPPE is designed to deal with low SNR datasets (infants and children EEG), while other methods were validated on adult EEG (where the data quality is comparatively better). We also highlight that it is the only algorithm that uses normalized power values (i.e., frequency domain) to detect bad channels. All other methods use time-series measures (e.g., Hurst Exponent, Pearson Correlation, Channel Variance). This observation suggests that the frequency-specific measure is more efficient in detecting artifacts at the channel level.

Given the outstanding performance of LOF, it is a promising bad channel detection in EEG acquired in any context from any population. In our previous work, we integrated LOF into NEAR, the artifact pipeline developed for newborn and infant EEG data [13]. Thanks to its high degree of versatility, LOF can also be integrated into other existing EEG artifact removal pipelines such as FASTER (for adult EEG) or HAPPE (for infant EEG) by replacing their respective bad channel techniques with LOF, which might lead to better overall artifact removal. We made the source code freely available as an EEGLAB plugin [38]. Even though we have not investigated the performance of LOF on Magneto-encephalography (MEG) data, we believe LOF can benefit MEG artifact removal as well.

## 6. Conclusions

In this work, we proposed an adaptable and robust EEG bad channel detection tool based on the Local Outlier Factor (LOF) algorithm. We demonstrated that LOF scores are less sensitive to outliers present in the data, thereby providing a better estimation of the outliers compared to existing measures used in the EEG literature. We validated our approach on real EEG acquired from three populations representative of different experimental designs and SNR ranges. This is the first study to validate bad channel detection methods on different population datasets. We showed that LOF is flexible to all kinds of EEG and outperforms the widely used SoA bad channel detection methods.

## Figures and Tables

**Table 1:** Summary of results on simulated EEG with 64 channels. ND = Not Detected; PD = Partially Detected; FD = Fully Detected.

Channel ID	1, 49	6, 35	16	False Positives	F1 Score
Methods | Kind of Artifacts	Flat Line	Motion	Aperiodic		
**Kurtosis**	PD	FD	FD	2	0.73
**FASTER**	ND	FD	ND	1	0.4
**CRD**	FD	FD	ND	0	0.89
**HAPPE**	FD	FD	ND	0	0.89
**LOF**	ND	FD	FD	0	0.75
**LOF + Flat Line Detector**	FD	FD	FD	0	1

**Table 2:** Summary of results on real EEG.

Data/Method	Mean F1 Score (s.e.m.)				
	Kurtosis	FASTER	CRD	HAPPE	LOF
**Newborn**	0.30 (0.022)	0.40 (0.014)	0.38 (0.019)	0.45 (0.016)	**0.63 (0.018)**
**Infant**	0.23 (0.012)	0.17 (0.011)	0.21 (0.006)	0.25 (0.008)	**0.35 (0.007)**
**Adult**	0.14 (0.008)	0.15 (0.006)	0.11 (0.008)	0.24 (0.006)	**0.45 (0.016)**

## Data Availability

A part of the newborn data used in this study is freely available at https://osf.io/79mzg/ (accessed on 11 August 2022) and adult data used in this study can be found at https://openneuro.org/datasets/ds002034/versions/1.0.3 (accessed on 11 August 2022).

## Appendix A. Simulating EEG Using SEREEGA Toolbox

In this work, we generated Steady-State Visually Evoked Potential (SSVEP) data with 64 channels using the toolbox SEREEGA [32]. There are three components in this process. First, a component containing SSVEP response is simulated. Second, a component containing background EEG activity is generated. These two components are combined using a defined Signal-to-Noise ratio. The third component contains the details of the added channel artifacts. Here, we provide the relevant code snippets.

Component 1: An SSVEP response with a stimulation frequency of 0.8 Hz was added in bilateral sources in the early visual cortex (MNI coordinates: [−8 −76 10] and [8 −76 10]). As the SEREEGA toolbox does not directly support SSVEP data simulation, we used the special feature within the toolbox that allows the inclusion of the existing time series. To do this, we first generated two symmetrical sources using the following MATLAB command:

1	% generate two symmetrical sources in the early visual cortex
2	source1 = lf_get_source_nearest(leadfield, [−8 −76 10]); %left hemisphere
3	source2 = lf_get_source_nearest(leadfield, [8 −76 10]); %right hemisphere
4	sourceV1=[source1 source2]; % combined

Then, we created a data class component that contains SSVEP signal of 0.8 Hz as follows:

1 SSVEP = struct(); % empty struct
2 SSVEP.data = sin(2*pi*0.8*t); % 0.8 Hz sinusoidal signal
3 SSVEP.index = {‘e’, ‘:’};
4 SSVEP.amplitude = 0.5; % this value is derived from a real newborn EEG dataset
5 SSVEP.amplitudeType = ‘relative’;

We generated the SSVEP component using the SEREEGA function *utl_create_component* as follows:

1 SSVEP_component = utl_create_component(sourceV1, SSVEP, leadfield);
2 SSVEP_scalp = generate_scalpdata(SSVEP_component, leadfield, config); % ... scalp EEG is generated

Component 2: Event-unrelated ongoing EEG activity was generated in 62 randomly selected cortical sources, plus in the 2 sources of the first component located in the early visual cortex.

To simulate background EEG activity, we first generated 62 noise sources and projected the Brown noise across all those sources using the same function *utl_create_component*.

1	% generate 62 sources of noise in random voxels
2	noise_source = lf_get_source_spaced(leadfield, 62, 25);
3	noise_signal = struct(‘type’, ‘noise’, ‘color’, ‘brown’, ‘amplitude’, 1);
4	noise_components = utl_create_component([noise_source sourceV1], ... noise_signal, leadfield);
5	noise_scalp = generate_scalpdata(noise_components, leadfield, config);

Then, Component 1 (SSVEP) and Component 2 (Background noise) were mixed using the function *utl_mix_data* for a defined SNR as follows:

1 signal_scalp = utl_mix_data(SSVEP_scalp, noise_scalp, snr); % SSVEP and ... background EEG are mixed

Component 3: Once the neural signal was generated, artifacts in five randomly chosen channels were added by generating flat signals, motion noise, and jump-like artifacts.

To generate flat signals, we assigned a constant amplitude for channels 1 and 49. To generate motion noise on single channels, we designed a bandpass filter (0–10 Hz passband) and superimposed the filtered signals with original signals at random time intervals. Likewise, to create the jump-like artifact, we generated a sawtooth signal (MATLAB [40]) and superimposed it at random time points with the original signal.
